# Chalcone-containing dual-targeting PD-L1/tubulin small molecules: a novel approach for cancer immunotherapy

**DOI:** 10.3389/fphar.2026.1740903

**Published:** 2026-01-14

**Authors:** Yingxing Zhou, Jingwei Ding, Shayu An, Lu Wang, Xinyi He, Jingjing Du, Zhenhong Su, Xiao Yao

**Affiliations:** 1 School of Medicine, Hubei Polytechnic University, Huangshi, Hubei, China; 2 Hubei Key Laboratory for Kidney Disease Pathogenesis and Intervention, Hubei Polytechnic University, Huangshi, Hubei, China; 3 Hubei Provincial Engineering Research Center of Immunotherapy Drugs for Renal Tumors, Hubei Polytechnic University, Huangshi, Hubei, China; 4 Huangshi Key Laboratory of Molecular Diagnostics and Personalized Therapy, Huangshi Loveandhealth Hospital Affiliated Of Hubei Polytechnic University, Huangshi, Hubei, China; 5 Wuchang Hospital Affiliated to Wuhan University of Science and Technology, Wuhan Wuchang Hospital, Wuchang, China

**Keywords:** cancer immunotherapy, chalcone, dual targeting, PD-1/PD-L1, tubulin

## Abstract

**Objectives:**

This study aims to identify novel small-molecule inhibitors capable of dual targeting of PD-L1 and tubulin, intending to enhance cancer immunotherapy.

**Methods:**

A combination of computer-aided virtual screening, molecular docking, homogeneous time-resolved fluorescence (HTRF) assays, tubulin polymerization inhibition assays, and *in vivo* antitumor assays was utilized to identify compounds with dual-targeting potential.

**Results:**

Compound PP-1 exhibited moderate inhibitory activity against the PD-1/PD-L1 interaction (IC_50_ = 81.1 µM) and showed dose-dependent inhibition of tubulin polymerization (IC_50_ = 70.1 µM). Molecular docking analysis further confirmed that PP-1 can effectively bind to both PD-L1 and tubulin at the molecular level, supporting its bifunctional targeting capability. Importantly, compound PP-1 (50 mg/kg, P.O.) demonstrated significant antitumor efficacy in a melanoma model, achieving a tumor growth inhibition rate of 42% without apparent systemic toxicity.

**Conclusion:**

PP-1 demonstrates dual-target inhibitory activity against both the PD-1/PD-L1 immune checkpoint and tubulin polymerization, underscoring its potential as a promising lead compound for the development of next-generation dual-functional anticancer agents.

## 1 Introduction

**FIGURE 1:**
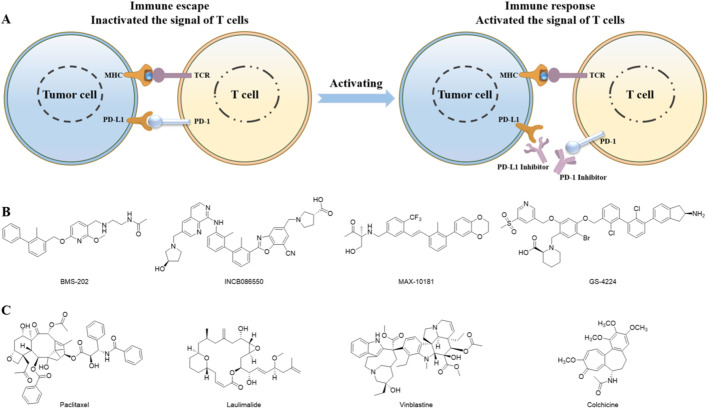
**(A)** Schematic diagram of the mechanism for the inhibition of PD-1/PD-L1 pathway in cancer treatment; **(B)** structurally diverse small molecules targeting PD-1/PD-L1 pathway; **(C)** Representative tubulin-targeted small molecule inhibitors.

Cancer is a leading cause of mortality worldwide, presenting major public health challenges (Alsina et al., 2023). Cancer immunotherapy plays a pivotal role in malignancy treatment and has shown substantial therapeutic benefits (Boisgerault and Bertrand, 2023; Brahmer et al., 2023). As one of the most promising research directions in oncology, immunotherapy continues to revolutionize cancer treatment (Choueiri et al., 2023; Chow et al., 2022). In clinical practice, immunotherapy predominantly relies on macromolecular drugs such as monoclonal antibodies like Opdivo and Keytruda (Dai et al., 2022). However, these antibody-based therapies face several limitations, including but not limited to low oral bioavailability and a prolonged metabolic half-life, which may lead to immune-related adverse reactions (irAEs) (Cheng et al., 2018). In contrast, small-molecule compounds offer distinct pharmacodynamic advantages, such as enhanced oral absorption and cost-effective manufacturing, making them a focal point in contemporary cancer immunotherapy research (Lin et al., 2008; Peng et al., 2023; Qin et al., 2020; Qin et al., 2019). The PD-1/PD-L1 axis represents a druggable therapeutic target in cancer immunotherapy (Skalniak et al., 2017; Su et al., 2023). Mechanistically, the interaction between PD-1 and PD-L1 induces downregulation of T-cell effector functions in cancer patients, thereby suppressing anti-tumor immune responses (Figure 1A). PD-1/PD-L1 inhibitors counteract this immunosuppressive mechanism by blocking ligand-receptor binding, thus restoring immune activation (Sun et al., 2022; Wang T. et al., 2021; Wang Y. et al., 2021; Yi et al., 2022). Currently, several small-molecule PD-L1 inhibitors, including INCB086550, MAX-10181, and GS-4224, are under clinical investigation (Figure 1B). These compounds share structural similarities with BMS-202, featuring a characteristic biphenyl scaffold. However, clinical studies have demonstrated that PD-L1 inhibitors as monotherapy exhibit limited efficacy. Additional research into novel combination therapies is essential to further enhance the efficacy of immunotherapy.

Microtubules, as fundamental structural components of cellular architecture, represent one of the most effective therapeutic targets in cancer treatment. Colchicine-binding site inhibitors (CBSIs) demonstrate significant potential in developing next-generation antimitotic agents, as they generally exhibit reduced susceptibility to drug resistance caused by overexpression of drug efflux pumps, thereby positioning CBSIs as promising candidates for novel tubulin inhibitors (Peng et al., 2022). Tubulin inhibitors can be classified into several categories based on structural characteristics: colchicine derivatives, CA-4 analogues, chalcone analogues, coumarin analogues, indole hybrids, quinoline and quinazoline analogues, lignan and podophyllotoxin derivatives, phenothiazine analogues, *N*-heterocyclic compounds, and others. Most of these compounds have demonstrated potent antitumor activity in both *in vitro* and *in vivo* studies, including antiproliferative effects against multidrug-resistant (MDR) cell lines and anti-angiogenic properties (Figure 1C). Recently, clinical studies have demonstrated that the combination of PD-1/PD-L1 antibodies with tubulin inhibitors exerts synergistic effects, achieving enhanced antitumor efficacy (Katsarou et al., 2022; Liu et al., 2023; Maliekal et al., 2022; Yang et al., 2021). This may result from tubulin inhibitors facilitating tumor antigen release, providing damage-associated molecular patterns (DAMPs) during apoptosis induction, promoting cytotoxic CD8^+^ T cell activation, and increasing tumor cell sensitivity to PD-1/PD-L1 blockade. However, such combination therapies present inherent limitations, including unpredictable pharmacokinetic (PK) and pharmacodynamic (PD) profiles (de Visser and Joyce, 2023; Dexheimer et al., 2023). Dual-target small-molecule inhibitors simultaneously addressing PD-L1 and tubulin offer an attractive therapeutic strategy due to their controllable PK/PD characteristics. In this work, we identified several PD-L1 and Tubulin dual-functional small-molecule inhibitors through computer-aided drug virtual screening, homogeneous time-resolved fluorescence (HTRF) target assays, and tubulin inhibition activity evaluation. These findings provide valuable insights and strategic directions for developing next-generation PD-L1-based dual-functional inhibitors.

## 2 Materials and methods

### 2.1 Computer-aided virtual screening and molecular docking experiments

The virtual screening compounds were sourced from the TargetMol Natural Compound Library. The crystal structure of PD-L1 protein bound to BMS-202 (PDB code: 5J89) was selected as the structural model for virtual screening and molecular docking. This choice is justified by the structure’s high resolution (2.20 Å): it ensures atomic-level accuracy of PD-L1’s binding pocket, thereby supporting the reliability of subsequent docking analyses. The LigPrep module was employed for ligand preparation, followed by molecular docking to the BMS-202 binding site. The top-ranked docking complexes underwent energy minimization of structural strains via restrained molecular dynamics simulations. During optimization, the ligand and residues within a 15 Å radius were allowed free movement, while residues beyond this radius remained rigidly fixed. In the virtual screening phase, a total of 1,867 molecules were selected from the natural products (NP) subset of Targetmol’s compound library. The approximately 50 top-ranked compounds identified in the primary screening were further evaluated for druglikeness, medicinal chemistry features, and pharmacokinetic profiles. Five established drug-likeness filters—Lipinski’s Rule of Five, Veber’s Rule, Ghose Filter, Muegge Filter, and Egan Filter—were applied sequentially. Only compounds that met all five criteria, receiving positive assessments across the board, were retained for downstream analysis. Compounds predicted to exhibit high oral absorption were prioritized, whereas pan-assay interference compounds (PAINS) were excluded to minimize the risk of false-positive outcomes. The AMES toxicity and carcinogenicity potential of the remaining candidates were assessed using ADMETlab 3.0. Those with predicted scores below 0.3—classified as AMES-negative and non-carcinogenic—were considered to possess favorable safety profiles. Following this comprehensive, multi-stage screening process, four compounds were ultimately selected and are presented in this study.

### 2.2 *In Vitro* PD-1/PD-L1 binding assay

The inhibitory effects of the virtually screened compounds on PD-1/PD-L1 interaction were evaluated using a PD-1/PD-L1 HTRF (Homogeneous Time-Resolved Fluorescence) binding assay. The PD-1/PD-L1 Binding Assay Kit (Catalog No.: 64PD1PEG) was purchased from PerkinElmer, and experiments were conducted according to the manufacturer’s protocol. Briefly, the interaction between Tag1-PD-L1 and Tag2-PD-1 was assessed using anti-Tag1-Europium (HTRF donor) and anti-Tag2-XL665 (HTRF acceptor) in a homogeneous time-resolved fluorescence (HTRF) assay. The experiment included a negative control group, a positive control group, and a treatment group, with three replicate wells per group. For the negative control group, 2 μL of diluent buffer, 4 μL of Tag1-PD-L1 diluted in diluent buffer, and 4 μL of Tag2-PD-1 diluted in diluent buffer were sequentially added to each well of a 384-well plate. For the treatment group, 2 μL of test compound diluted in diluent buffer was added to each well, followed by the addition of 4 μL of Tag1-PD-L1 and 4 μL of Tag2-PD-1, both diluted in diluent buffer. After sample addition across all groups, the plate was incubated at 37 °C for 15 min. Subsequently, 5 μL of detection reagent—containing anti-Tag1-Eu^3+^ and anti-Tag2-XL665 diluted in detection buffer—was added to each well. The plate was then incubated at 37 °C for 1 h to overnight, after which fluorescence intensities at 665 nm and 620 nm were measured using a PerkinElmer Envision multimode microplate reader. The HTRF ratio was calculated as (665 nm/620 nm) × 10^4^. Each compound was tested across 8–10 concentration points to calculate IC_50_ values.

### 2.3 *In Vitro* tubulin polymerization assay

Porcine brain tubulin was tested in a 100 mM PIPES buffer (pH 6.5) containing 1 mM MgSO_4_, 2 mM EGTA, 1 mM GTP, and 1 mM 2-mercaptoethanol. Three temperature-dependent assembly/disassembly cycles were performed for purification. During the first polymerization cycle, glycerol and phenylmethylsulfonyl fluoride (PMSF) were added to final concentrations of 4 M and 0.2 mM, respectively. Homogeneous tubulin was isolated via phosphocellulose chromatography and aliquoted for storage at −70 °C. For the assay, tubulin was mixed with test compounds at varying concentrations in PEM buffer (100 mM PIPES, 1 mM MgCl_2_, and 1 mM EGTA) containing 5% glycerol at 0 °C for 5 min. Subsequently, 1 mM GTP was added to the reaction mixture. Microtubule polymerization was monitored at 37 °C by measuring light scattering at 340 nm using a SPECTRA MAX 190 spectrophotometer (Molecular Devices). Data were quantified based on plateau absorbance values.

### 2.4 Immunofluorescence staining

HepG2 cells were seeded on glass coverslips in 24-well plates. After 12 h of drug treatment, the culture medium was aspirated, and cells were fixed with 4% paraformaldehyde, followed by permeabilization with phosphate-buffered saline (PBS) containing 0.2% Triton X-100. Cells were blocked with 100 µL goat serum albumin at room temperature for 30 min, then incubated with a monoclonal antibody (anti-β-tubulin) at 37 °C for 1 h. After three PBS washes, secondary antibody staining was performed. Nuclei were counterstained with 4′,6-diamidino-2-phenylindole (DAPI), and cellular morphology was visualized using fluorescence microscopy.

### 2.5 PD-1/PD-L1 NFAT reporter bioassay

A PD-1/PD-L1-dependent NFAT transcriptional reporter system was established to assess inhibitor efficacy using engineered Jurkat T cells (PD-1/NFAT/Jurkat) and CHO-K1 effector cells (CHO/PD-L1/TCR). The Jurkat reporter line stably expresses PD-1 and an NFAT promoter-driven luciferase construct, while CHO effector cells co-express PD-L1 and TCR-activating ligands. Before co-culture, CHO/PD-L1/TCR cells were plated in 96-well microplates at 3.5 × 10^5^ cells/mL in RPMI 1640 complete medium and equilibrated for 24 h under standard culture conditions (37 °C, 5% CO_2_). Following supernatant removal, serial compound dilutions were applied to effector cells for 30 min pre-incubation. PD-1/NFAT/Jurkat cells (4 × 10^5^ cells/mL) were subsequently introduced to initiate ligand-receptor interaction. Post 5 h co-culture under physiological conditions, plates were equilibrated to ambient temperature for 10 min prior to luminescent substrate addition. Bio-GloTM Luciferase Reagent (100 μL/well) was incubated with cell lysates for 10 min, followed by photon quantification using a FlexStation 3 multimode microplate reader (Molecular Devices, San Jose, CA).

### 2.6 Molecular docking

The crystal structures of PD-L1 and Tubulin were downloaded from the protein data bank (www.rcsb.org) and were prepared by adding hydrogen atoms and removing the water molecules. The protein structure was minimized using the OPLS 2005 force field. The structure of PP-1 was constructed in Maestro and further processed with the LigPrep panel. The binding sites were set at a 30Å*30Å*30 Å box in the center of the initial compound.

### 2.7 *In vivo* efficacy study in B16-F10 tumor models

The animal protocols were approved by the National Institutional Animal Care and Ethical Committee at Hubei Polytechnic University and followed the Guide for the Care and Use of Laboratory Animals. 6–8 weeks-old male BALB/c mice were purchased from Liaoning Changsheng Biotechnology Co., Ltd. A total of 1 × 10^5^ B16-F10 melanoma cells were inoculated into the right flank of each mouse according to the protocols of tumor transplant research. After the tumor size reached approximately 50 mm^3^ in volume, the mice were randomly assigned to the vehicle control or treatment group. Tumor volume was measured every 2–3 days using a traceable electronic digital caliper, calculated as TV = (length × width^2^)/2 (mm^3^), and tumor growth inhibition (TGI) was determined by the formula: TGI (%) = [1 − (Wt/Wv)] × 100%, where Wt represents the final mean tumor weight of the treatment group and Wv denotes that of the vehicle control group. Harvested organs were fixed in 4% paraformaldehyde, routinely processed into paraffin sections, stained with hematoxylin and eosin (H&E), and imaged under a light microscope for pathological evaluation.

### 2.8 Flow cytometry

Mouse organs were collected, and tumor cells were mechanically isolated using a 40 μm cell strainer. Cells were stained at 4 °C for 30 min and flow cytometry (BD, United States) was performed using antibodies against the following targets (CD3, CD4, CD8, PD-L1) and isotype controls, all provided by BioLegend company: FITC anti-mouse CD3 Antibody (Cat No:100204, LOT: B249620), APC anti-mouse CD8a Antibody (Cat No:100711, LOT: B280032), Anti-mouse-PD-L1-PE Antibody (Cat#:124307, Lot: B284420), Rat IgG2b K Isotype Control PE (Cat#:400608, Lot: B156144).

## 3 Results

The PD-L1 co-crystal structure containing ligands (PDB code: 5J89) was applied for molecular virtual screening, as its high resolution of 2.20 Å ensures atomic-level accuracy of PD-L1’s binding pocket and thereby supports the reliability of subsequent docking analyses (compound library, TargetMol Chemicals Inc.). Subsequently, top-ranked compounds identified through high-throughput virtual screening were evaluated for their inhibitory effects on PD-1/PD-L1 interaction using a well-established homogeneous time-resolved fluorescence (HTRF) assay. The virtual screening results and corresponding activity data of the prioritized compounds are summarized in Table 1, with BMS-202 (CAS No.: 1675203–84-5, IC_50_ = 25 nM), developed by Bristol-Myers Squibb (BMS), serving as the positive control in the HTRF assay. As shown in Table 1, compounds with high docking scores exhibited moderate inhibitory activity against the PD-1/PD-L1 target in HTRF assays, with inhibition rates in the micromolar range. The most promising compound, PP-1, exhibited an acceptable IC_50_ value of 81.1 ± 8.6 µM against PD-1/PD-L1. Structural analysis of the active compounds revealed that these molecules share a common chalcone-derived core scaffold. Chalcones, a class of polyphenolic compounds widely found in medicinal plants, feature an α,β-unsaturated ketone moiety linking two aromatic rings. This flexible framework enables diverse receptor interactions, contributing to their broad biological activities, including anti-inflammatory, antioxidant, and antitumor effects. Additionally, chalcone derivatives are advantageous due to their structural simplicity, ease of synthesis, and amenability to modification. Structural optimization based on natural product scaffolds represents a key strategy for developing novel drugs or lead compounds (Rudrapal et al., 2021; Zhuang et al., 2017). In this work, high-throughput virtual screening identified a novel class of PD-1/PD-L1 inhibitors with novel chalcone-based structures, which serve as promising lead candidates for further structural refinement to enhance potency and selectivity.

**TABLE 1:** PD-1/PD-L1 inhibitors identified from virtual screening.

Structures	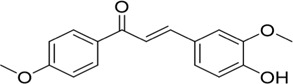	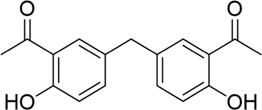	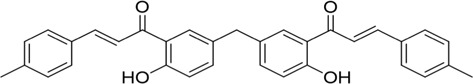	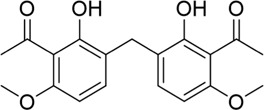
ID	PP-1	PP-2	PP-3	PP-4
Scoring values	−8.9 kcal/mol	−7.2 kcal/mol	−8.3 kcal/mol	−7.1 kcal/mol
PD-1/PD-L1 IC_50_	(81.1 ± 8.6) μM	>100 µM	(94.6 ± 11.3) µM	>100 µM

**FIGURE 2:**
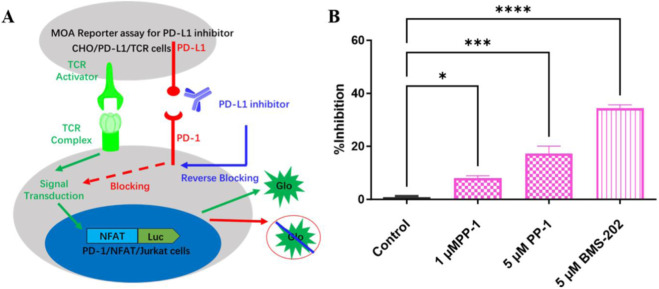
**(A)** Principle of the reporter assay for anti-PD-1/PD-L1 inhibitors; **(B)** Activities of PP-1 and BMS-202 in the PD-1/PD-L1 NFAT reporter model. *P < 0.05, **P < 0.01, ***P < 0.001, ****P < 0.0001, One-way analysis of variance (ANOVA).

Having identified the potent PD-L1-inhibitory activity of PP-1, we explored the immunomodulatory ability of PP-1 in a cell-based PD-1/PD-L1 NFAT reporter bioassay. As shown in Figures 2A,B, Compound PP-1 demonstrated the ability to inhibit PD-1/PD-L1 interaction. In the presence of 5 μM PP-1, PD-1/PD-L1 inhibition was increased by 17.7%, verifying that the compound PP-1 can effectively block the PD-1/PD-L1 interaction and enhance the anti-tumor immunotherapy efficacy.

**FIGURE 3:**
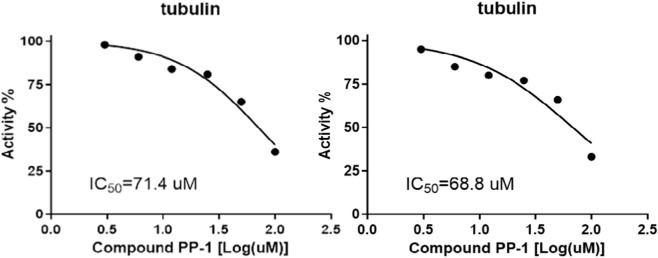
Inhibition of tubulin polymerization by PP-1.

Tubulin plays a pivotal role in the proliferation of tumor cells, making it an ideal target for anticancer therapeutics. As previously discussed, chalcone-derived core scaffolds exhibit potential inhibitory activity against tubulin. To investigate the inhibitory effects of compound PP-1 on tubulin, we conducted a tubulin polymerization assay. As shown in Figure 3, PP-1 demonstrated significant inhibition of microtubule polymerization (IC_50_ = 70.1 µM). Microtubules, as critical components of the cytoskeleton, are essential for regulating cellular architecture, signal transduction, mitosis, and motility. Targeting microtubule dynamics has emerged as a successful chemotherapeutic strategy (Xiao et al., 2021). The tubulin polymerization assay revealed that PP-1 effectively disrupted microtubule assembly in a dose-dependent manner across the concentration range of 3–100 μM (Figure 3), confirming its dual functionality as a tubulin inhibitor.

**FIGURE 4:**
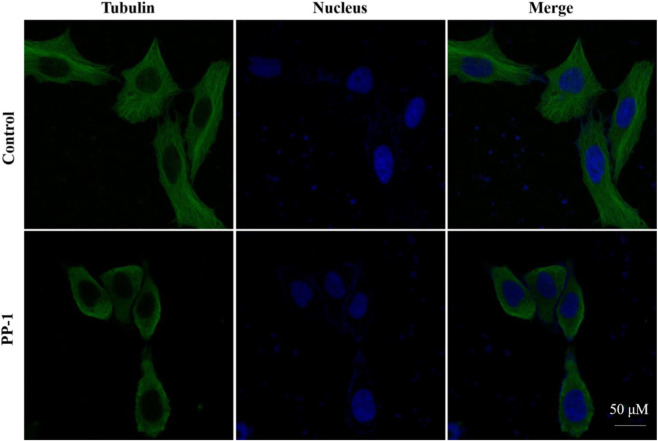
Effects of PP-1 on microtubules. HepG2 cells were treated with vehicle control 0.1% DMSO, PP-1 (10 µM) for 12 h. Microtubules were visualized with an anti-tubulin antibody (green), and the cell nucleus was visualized with DAPI (blue). Fluorescence images were collected by an LSM 880 laser confocal microscope (Carl Zeiss, Germany).

To further investigate the cellular-level effects, we evaluated PP-1’s inhibitory activity on microtubule networks in HepG2 cells. As illustrated in Figure 4, treatment with PP-1 at a 10 µM concentration resulted in significant disruption of microtubule architecture in HepG2 cells, demonstrating that PP-1 effectively engages the tubulin target at the cellular level.

**FIGURE 5:**
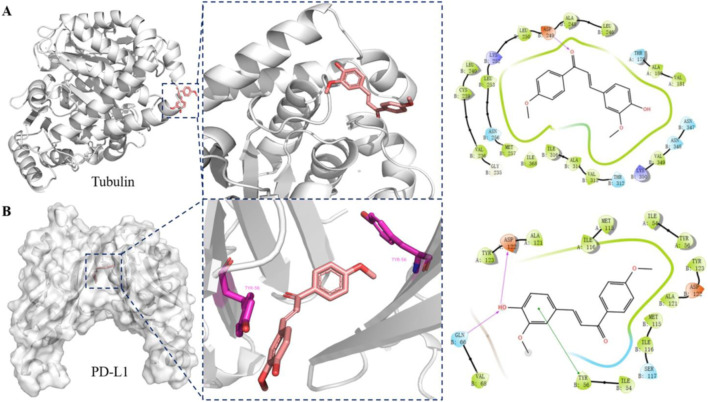
Binding interactions between compound PP-1 and target proteins (tubulin and PD-L1): **(A)** Docking analysis of compound PP-1 in the colchicine binding of tubulin (PDB: 5H7O); **(B)** Docking analysis of compound PP-1 in the hydrophobic cleft formed by the dimeric PD-L1 (PDB: 5J89).

To further elucidate the dual-target mechanism of compound PP-1 and its interactions with the target proteins, we performed molecular docking simulations of PP-1 with both PD-L1 and tubulin. As shown in Figure 5A, PP-1 bound effectively to the colchicine-binding site of tubulin, forming a hydrogen bond with the Asp249 residue. The molecular docking results align with the tubulin polymerization assay data (IC_50_ = 70.1 µM), demonstrating that PP-1 inhibits microtubule assembly by directly engaging tubulin. Furthermore, PP-1 exhibited strong binding to PD-L1, forming a key π-π interaction with Tyr56 residues. In addition to the key π-π interaction between PP-1 and Tyr56 of PD-L1, our molecular docking results further demonstrate that PP-1 also forms two hydrogen bonds with Gln66 and Asp122 of PD-L1—all of which collectively contribute to enhancing its binding affinity to PD-L1 and thus underpin its potent activity. (Figure 5B). These interactions stabilized the PP-1/PD-L1 complex, consistent with its observed inhibitory activity in the HTRF assay (IC_50_ = 81.1 ± 8.6 µM). Collectively, these findings validate PP-1 as a dual-functional small molecule capable of molecular-level interactions with both PD-L1 and tubulin, supporting its dual-target inhibitory mechanism.

**FIGURE 6:**
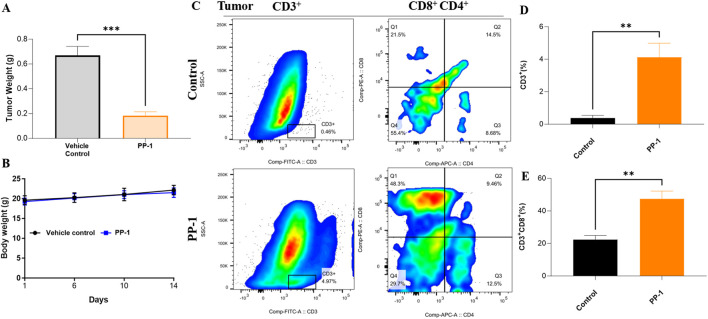
*In vivo* efficacy of compound PP-1 was evaluated in the B16-F10 melanoma tumor model established in BALB/c mice. **(A)** Tumor weight; **(B)** Body weight changes. **(C)** Proportions of tumor-infiltrating lymphocytes (TILs) in B16-F10 tumors. **(D,E)** Statistical analysis of TIL frequencies. Data are presented as mean ± SD, *P < 0.05, **P < 0.01, ***P < 0.001, ****P < 0.0001, Student’s t-test.

To further evaluate the *in vivo* anti-tumor efficacy of compound PP-1, we assessed its antitumor activity in the mouse B16-F10 melanoma model. As illustrated in Figures 6A,B, oral administration of PP-1 at a dose of 50 mg/kg significantly suppressed the growth of B16-F10 melanoma tumors, achieving a tumor inhibition rate of 42%, as compared to the vehicle control group. Moreover, the body weight of the treated mice remained stable throughout the experimental period, suggesting that PP-1 exhibits favorable *in vivo* safety. To further investigate its immunomodulatory effects, we analyzed the infiltration levels of CD3^+^ and CD8^+^ T cells within the tumor microenvironment. Flow cytometry results revealed a marked increase in the frequency of CD3^+^ cells (4.12% vs. 0.39%) and CD3^+^CD8^+^ cells (47.5% vs. 22.3%) in the PP-1 treatment group compared to the vehicle control group (Figures 6C–E). Collectively, these findings indicate that PP-1 effectively modulates the tumor immune microenvironment by promoting the activation and infiltration of effector T-cell subsets, thereby enhancing antitumor immune responses.

**FIGURE 7:**
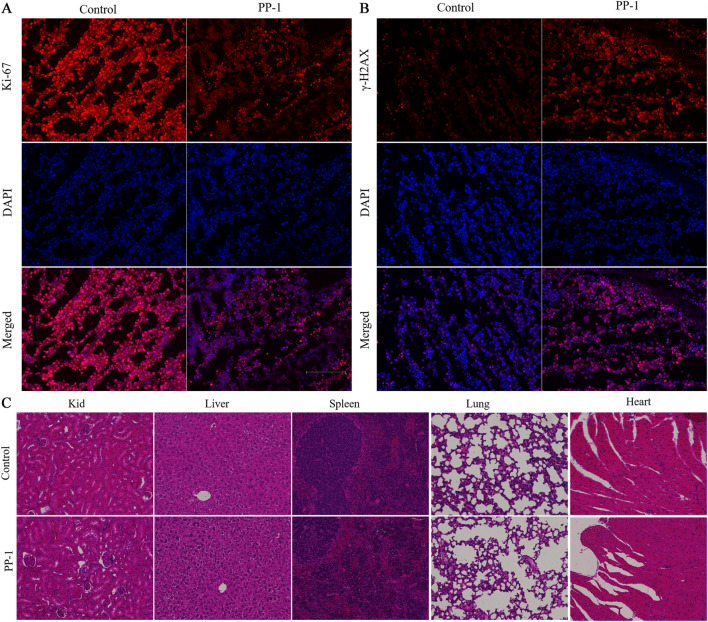
Immunofluorescence images of Ki-67 **(A)** and γ-H2AX **(B)** in tumor tissues treated with vehicle and PP-1; **(C)** Pathological analysis of tissue sections from major organs (heart, liver, kidney) of melanoma tumor-bearing mice. Hematoxylin and eosin (H&E) were used to stain the organs, with representative images captured.

To further elucidate the mechanism by which compound PP-1 enhances antitumor immunity, we performed immunofluorescence staining to evaluate the impact of PP-1 on the expression of Ki-67 (a marker of cell proliferation) and γ-H2AX (a well-established biomarker of DNA double-strand breaks) in B16-F10 melanoma tissues. As shown in Figure 7A, the PP-1 treatment group exhibited a marked reduction in Ki-67 expression compared to the vehicle control. Notably, γ-H2AX signals were significantly increased in the PP-1-treated group, indicating robust induction of DNA damage (Figure 7B). These results demonstrate that PP-1, as a tubulin/PD-L1 dual inhibitor, exerts dual antitumor effects: it suppresses tumor cell proliferation and induces DNA double-strand breaks (DSBs), leading to direct tumor cell death. This cytotoxic effect promotes the release of tumor-associated neoantigens, thereby reshaping the tumor immune microenvironment by converting immunologically “cold” tumors into “hot” tumors with enhanced immune cell infiltration and activation. To evaluate the *in vivo* safety profile of compound PP-1, major organs, including the liver, kidney, and heart, were collected from treated mice and subjected to hematoxylin and eosin (H&E) staining for histopathological examination. As shown in Figure 7C, no apparent morphological abnormalities were detected in tissues from mice receiving PP-1 therapy, suggesting a favorable safety profile for PP-1.

## 4 Discussion

Cancer has emerged as the second leading cause of death globally and a major public health challenge. Current molecular-targeted therapies aim to selectively engage single biological targets to avoid off-target effects. However, cancer is a complex disease characterized by multiple dysregulated signaling pathways, rendering single-target agents are insufficient for achieving therapeutic efficacy. This limitation has driven interest in multi-target therapeutics. Compared to single-target drugs, multi-target agents can simultaneously modulate multiple pathways, thereby enhancing therapeutic outcomes and reducing the likelihood of drug resistance. Recent studies demonstrate that combining PD-1/PD-L1 antibodies with tubulin inhibitors such as paclitaxel achieves synergistic antitumor effects, with combination regimens showing improved safety profiles compared to monotherapies (Fong et al., 2019; Ren et al., 2023). Nevertheless, combination therapies face inherent challenges, including unpredictable pharmacokinetic (PK) and pharmacodynamic (PD) interactions when multiple drugs are co-administered. Consequently, designing single-molecule agents capable of engaging multiple validated anticancer targets has become a key focus in multi-target drug discovery, as single-molecule PK/PD profiles are more predictable and easier to optimize.

## 5 Conclusion

In this study, we employed computer-aided drug design (CADD) strategies to identify a novel dual-target inhibitor (PP-1) with activity against both PD-L1 and tubulin. Mechanistic investigations revealed that PP-1 disrupts PD-1/PD-L1 binding (IC_50_ = 81.1 ± 8.6 µM) and inhibits tubulin polymerization (IC_50_ = 70.1 µM). The B16-F10 tumor-bearing mouse experiments demonstrated that PP-1 effectively suppressed tumor growth while maintaining good *in vivo* safety. These findings position PP-1 as a promising starting point for developing more potent dual inhibitors of tubulin polymerization and PD-1/PD-L1 interaction. Its dual-target mechanism highlights the potential for advancing PP-1 as a novel cancer immunotherapeutic agent targeting both PD-L1 and tubulin. In our follow-up studies, we plan to conduct structural derivatization of PP-1’s scaffold to investigate the impact of key functional groups on its biological activity and establish a systematic structure-activity relationship (SAR), with the relevant findings to be reported in due course.

## Data Availability

The data are available from the corresponding authors on reasonable request.
